# Transplacental murine cytomegalovirus infection in the brain of SCID mice

**DOI:** 10.1186/1743-422X-4-26

**Published:** 2007-03-09

**Authors:** Nigel K Woolf, Dawn V Jaquish, Fred J Koehrn

**Affiliations:** 1 Departments of Surgery/Anatomy, University of California Medical School at San Diego Life, 9500 Gilman Drive, La Jolla, CA 92093-0604, USA; 2 Department of Surgery, Veterans Affairs Research Service, VA San Diego Healthcare System, 3350 La Jolla Village Drive, La Jolla, CA 92161, USA

## Abstract

**Background:**

Congenital cytomegalovirus (CMV) infection is the most common congenital viral infection in humans and the major nonhereditary cause of central nervous system (CNS) developmental disorders. Previous attempts to develop a murine CMV (MCMV) model of natural congenital human CMV (HCMV) infection have failed because MCMV does not cross the placenta in immunocompetent mice.

**Results:**

In marked contrast with immunocompetent mice, C.B-17 SCID (severe combined immunodeficient) mice were found to be highly susceptible to natural MCMV transplacental transmission and congenital infection. Timed-pregnant SCID mice were intraperitoneally (IP) injected with MCMV at embryonic (E) stages E0-E7, and vertical MCMV transmission was evaluated using nested polymerase chain reaction (nPCR), in situ hybridization (ISH) and immunohistochemical (IHC) assays. SCID mouse dams IP injected at E0 with 10^2 ^PFU of MCMV died or resorbed their fetuses by E18. Viable fetuses collected at E18 from SCID mice IP injected with 10^2^–10^4 ^PFU of MCMV at E7 did not demonstrate vertical MCMV transmission. Notably, transplacental MCMV transmission was confirmed in E18 fetuses from SCID mice IP injected with 10^3 ^PFU of MCMV at stages E3-E5. The maximum rate of transplacental MCMV transmission (53%) at E18 occurred when SCID mouse dams were IP injected with 10^3 ^PFU of MCMV at E4. Congenital infection was confirmed by IHC immunostaining of MCMV antigens in 26% of the MCMV nPCR positive E18 fetuses. Transplacental MCMV transmission was associated with intrauterine growth retardation and microcephaly. Additionally, E18 fetuses with MCMV nPCR positive brains had cerebral interleukin-1α (IL-1α) expression significantly upregulated and cerebral IL-1 receptor II (IL-1RII) transcription significantly downregulated. However, MCMV-induced changes in cerebral cytokine expression were not associated with any histological signs of MCMV infection or inflammation in the brain.

**Conclusion:**

Severe T- and B-cell immunodeficiencies in SCID mice significantly enhance the rate of natural MCMV transplacental transmission and congenital infection. During gestation MCMV exhibits a tissue tropism for the developing brain, and vertical MCMV transmission is correlated with fetal growth retardation and abnormal cerebral proinflammatory cytokine expression. These data confirm that natural vertical MCMV infection in SCID mice constitutes a useful new experimental rodent model of congenital HCMV infection.

## Background

Cytomegalovirus (CMV), a double-stranded DNA β-herpesvirus, is the most common cause of human congenital infection, with a prevalence rate of approximately 1% for all live births in the United States (worldwide range 0.5–2.4%) [1]. CMV is also the leading viral cause of disease morbidity and mortality in congenitally infected fetuses and premature neonates [2]. While the majority of congenital human CMV (HCMV) infections are asymptomatic, it has been estimated that 5–10% of HCMV congenitally infected neonates exhibit symptomatic, generalized cytomegalic inclusion disease (CID) [3]. Typical clinical features of CID include petechiae, hepatosplenomegaly, jaundice and microcephaly [4]. Additional manifestations of CID at birth include intrauterine growth retardation (IUGR), prematurity, chorioretinitis, and central nervous system (CNS) diseases such as intracranial calcifications, ventriculomegaly, lissencephaly, pachygyria, dysmyelination, paraventricular cysts and calcifications [5,6]. Up to 25% of neonates with CID die from disease complications, and more than 90% of survivors experience significant and permanent CNS and sensory impairments, including intellectual, motor, auditory and visual system deficits [4,7-9]. Notably, 90% of congenital HCMV infected neonates are asymptomatic at birth, but later in life develop significant sensory system disorders. The most prevalent delayed-onset sensory system sequelae of congenital HCMV infection is sensorineural hearing loss (SNHL), and at least 25% of children with congenital CMV-induced auditory deficits develop their hearing losses only after the first year of life [10-13]. It has been estimated that the direct and indirect costs for treating the sequelae of congenital HCMV infections exceeds $1.9 billion per year [14].

The pathogenesis of congenital HCMV-induced nervous system pathologies is still poorly understood. Because CMV exhibits strict species-specificity [2,15], it has not been possible to use HCMV to create experimental animal models of congenital HCMV infection. Experimental models of natural, transplacental congenital CMV infection have been developed in immunocompetent animals using species-appropriate guinea pig CMV (GPCMV) [16-26], rat CMV [27], pig [28] and rhesus macaque monkey CMV (RhCMV) [29,30]. Unfortunately, none of these animal models have been shown to induce the CNS and sensory system infections and pathologies observed in congenital HCMV infections [31].

Although immunocompetent mice are highly resistant to transplacental congenital MCMV infection [31,32], it has been reported that transplacental passage of lactate dehydrogenase-elevating virus (LDEV) was significantly enhanced in severe T- and B-cell immunodeficient SCID (severe combined immunodeficient) mice [33]. Consequently, we hypothesized that SCID mice might also be more vulnerable to transplacental MCMV transmission. Our results confirmed that pregnant SCID mice are highly susceptible to natural MCMV vertical transmission and congenital infection, and that MCMV transmitted across the placenta exhibits tissue tropisms for the developing fetal brain and viscera.

## Results

### Development of a SCID mouse model of congenital MCMV infection

SCID mice are extremely susceptible to MCMV infection, and even 1 PFU of MCMV will eventually kill these severely T- and B-cell immunodeficient animals [34]. The initial phase of this experiment consisted of a dose-mortality study conducted to test the susceptibility of SCID mice to congenital MCMV infection, and to establish an optimal experimental parameters for this model. SCID mouse dams were intraperitoneal (IP) injected with 10^2^–10^4 ^PFU of MCMV at developmental ages ranging from E0-E7, and then allowed to survive until stage E18 of the normal mouse 20 day gestational period. As shown in Table 1, none of the SCID mouse dams injected with 10^2 ^PFU of MCMV at E0 (N = 4) lived and sustained live fetuses at stage E18. In contrast, the majority of the SCID dams inoculated with 10^3 ^PFU of MCMV at E3-E7 lived and maintained live litters at stage E18. Notably, vertical transmission of MCMV was confirmed by nPCR amplification of MCMV immediate-early-gene-1 in live fetuses collected from SCID mouse dams inoculated with 10^3 ^PFU of MCMV at E3 (33%), E4 (100%) and E5 (25%), but not from dams similarly injected at E7 (0%) (Table 1). Given that all of the SCID mouse dams IP injected with MCMV at E3-E7 exhibited significant clinical signs of MCMV viremia at E18 (e.g., ruffled fur, weight loss, hunched posture, labored breathing and lethargy), nPCR negative littermates constituted an experimental control group which confirmed that fetal brains and abdominal viscera samples were not contaminated during tissue collection by maternal blood. Based on our findings that the maximum rate of fetal survival and transplacental MCMV transmission at E18 was obtained when SCID mouse dams were inoculated with 10^3 ^PFU of MCMV at developmental stage E4, we selected this experimental protocol for our subsequent studies.

**Table 1:** Transplacental MCMV transmission at E18 following intraperitoneal injection of SCID mouse dams.

**SCID DAM**	**PFU of MCMV**	**MCMV Injection Stage**	**Litter Size**	**nPCR Positive Fetuses**^a^
1	10^2^	E0	*	NA
2	10^2^	E0	*	NA
3	10^2^	E0	*	NA
4	10^2^	E0	NP	NA
5	10^3^	E3	*	NA
6	10^3^	E3	*	NA
7	10^3^	E3	NP	NA
8	10^3^	E3	NP	NA
9	10^3^	E3	5	1
10	10^3^	E3	6	1
11	10^3^	E4	6	1
12	10^3^	E4	4	4
13	10^3^	E4	3	2
14	10^3^	E4	8	1
15	10^3^	E5	NP	NA
16	10^3^	E5	7	0
17	10^3^	E5	6	0
18	10^3^	E5	6	4
19	10^2^	E7	NP	NA
20	10^2^	E7	7	0
21	10^2^	E7	5	0
22	10^3^	E7	6	0
23	10^4^	E7	NP	NA
24	10^4^	E7	NP	NA
25	10^4^	E7	NP	NA
26	10^4^	E7	NP	NA

**a: **nPCR MCMV DNA positive viscera; brains not tested

*: Dam died prior to E18

**NA: **Not applicable

**NP: **Not pregnant with live fetuses at E18

### Kinetics of MCMV transplacental transmission

**Figure 1:**
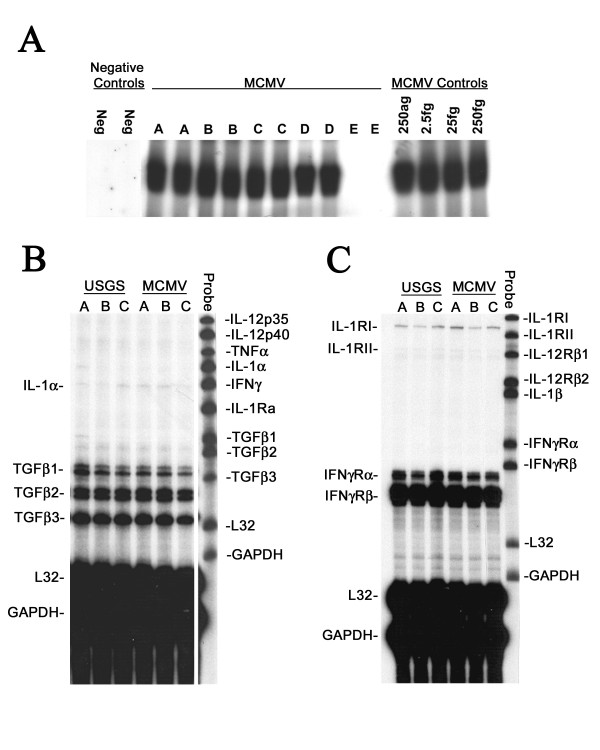
**Nested polymerase chain reaction (nPCR) and RNase protection assays (RPAs)**. **A:** Representative autoradiographs of liquid hybridizations run 2X following nested polymerase chain reaction (nPCR) assays for MCMV immediate-early gene 1 (Eco RI E) DNA in the viscera of E18 embryos from a C.B-17 SCID mouse injected with 10^3 ^PFU of MCMV at E4. Four embryos [lanes A-D] demonstrated MCMV amplification; the fifth fetus [lane E] was MCMV DNA negative. Negative control lanes contained AE elution buffer (see text). MCMV control lanes contained AE elution buffer and 250 ag-250 fg of MCMV DNA Eco RI E. Note that nPCRs exhibited saturation for the positive controls. **B-C: **Representative cytokine RPAs for the brains of E18 fetuses following maternal IP injection at E4 with uninfected salivary gland suspension (USGS) or 10^3 ^PFU of MCMV (MCMV). In each lane 10 μg of total brain mRNA from a single E18 fetus was hybridized with one of two probe sets designed to detect 10 proinflammatory cytokines and 6 corresponding cytokine receptor transcripts, as well as mL32 and GAPDH housekeeping controls (see text). Labeled probe sets were used as size markers in the right lane of each film.

We next investigated the kinetics of vertical MCMV transmission during gestation. SCID dams were IP injected with either uninfected salivary gland suspension (USGS) or 10^3 ^PFU of MCMV at developmental stage E4, and the fetuses collected at stages E12, E14, E16 or E18. None of the USGS control fetuses had MCMV DNA amplified by nPCR in their brains or viscera (Table 2). In marked contrast, MCMV DNA was detected by nPCR (Figure 1A) in fetuses at all of the postinfection stages examined (Table 3). Beginning as early as stage E12 (i.e., 8 days postinfection), MCMV DNA was amplified by nPCR in 38% of the fetuses (i.e., the whole body was assayed at E12 due to the small size of the fetus). By stage E14, the youngest gestational age at which the brains and viscera were examined separately, 21% of the fetuses had MCMV nPCR positive brains, and 50% amplified MCMV DNA in either their brains or viscera. The rate of CMV vertical transmission in the brain or viscera at stage E16 was 25%, and at E18 the incidence of transplacental MCMV transmission in reached a maximum of 53%. Thus, beginning as early as the end of the second trimester of pregnancy (i.e., E14), fetal SCID mouse brains and viscera were both major targets for vertical MCMV transmission.

**Table 2:** MCMV nPCR for fetuses from SCID mouse dams injected with USGS at E4.

**USGS Litters**				
**SCID DAM**	**Litters Positive/** **Total Litters**	**Brain**^+^**/** **Brains Tested**	**Viscera**^+^**/** **Viscera Tested**	**Brain**^+^** or Viscera**^+^/ **Fetuses Tested**

**E12**	0/4(0%)	*	*	0/32(0%)
**E14**	0/5(0%)	0/32(0%)	0/32(0%)	0/32(0%)
**E16**	0/6(0%)	0/19(0%)	0/19(0%)	0/19(0%)
**E18**	0/18(0%)	0/75(0%)	0/75(0%)	0/75(0%)

*: Whole body nPCR assayed to detect MCMV DNA at E12 due to the small size of the fetus.

**Table 3:** MCMV nPCR for fetuses from SCID dams injected with 10^3 ^PFU of MCMV at E4.

**E12 MCMV Litters**				
**SCID DAM**	**Litter Size**	**Negative Fetuses ** **(Brain**^-^**/Viscera**^-^**)**	**Positive Fetuses** ** (Brain**^+^** or Viscera**^+^**)**	**Brain**^+^** Fetuses ** **(Brain**^+^** or Viscera**^+^**)**^a^

1	4	4 (100%)	0 (0%)	*
2	7	7 (100%)	0 (0%)	*
5	4	4 (100%)	0 (0%)	*
4	9	2 (22%)	7 (78%)	*
5	5	1 (20%)	4 (80%)	*
Total	29	18 (62%)	11 (38%)	*

**E14 MCMV Litters**				

1	4	3 (75%)	1 (25%)	1 (25%)
2	3	0 (0%)	3 (100%)	1 (33%)
3	2	1 (50%)	1 (50%)	0 (0%)
4	8	2 (25%)	6 (75%)	3 (38%)
5	7	6 (86%)	1 (14%)	0 (0%)
Total	24	12 (50%)	12 (50%)	5 (21%)

**E16 MCMV Litters**				

1	5	4 (80%)	1 (20%)	1 (20%)
2	6	1 (16%)	5 (84%)	2 (33%)
3	6	5 (83%)	1 (17%)	0 (0%)
4	7	7 (100%)	0 (0%)	0 (0%)
5	4	4 (100%)	0 (0%)	0 (0%)
6	4	3 (75%)	1 (25%)	0 (0%)
Total	32	24 (75%)	8 (25%)	3 (10%)

**E18 MCMV Litters**				

1	8	7 (86%)	1 (14%)	0 (0%)
2	3	0 (0%)	3 (100%)	3 (100%)
3	5	0 (0%)	5 (100%)	3 (60%)
4	7	4 (57%)	3 (43%)	0 (0%)
5	7	2 (29%)	5 (71%)	1 (14%)
6	3	1 (33%)	2 (67%)	0 (0%)
7	5	3 (60%)	2 (40%)	1 (20%)
8	5	3 (60%)	2 (40%)	1 (20%)
9	3	0 (0%)	3 (100%)	1 (33%)
10	5	3 (60%)	2 (40%)	0 (0%)
11	5	2 (40%)	3 (60%)	0 (0%)
12	3	2 (67%)	1 (33%)	0 (0%)
13	10	7 (70%)	3 (30%)	0 (0%)
14	9	0 (0%)	9 (100%)	5 (56%)
15	10	7 (70%)	3 (30%)	0 (0%)
16	6	3 (50%)	3 (50%)	1 (17%)
Total	94	44 (47%)	50 (53%)	16 (17%)

*: Whole body nPCR assayed to detect MCMV DNA at E12 due to the small size of the fetus.

**a**: All nPCR brain^+ ^fetuses were also nPCR viscera^+^.

### Congenital MCMV infection and developmental pathology

The incidence of fetal demise was similar for both the USGS and MCMV groups (Table 4). Embryonic death *in utero *typically resulted in rapid resorption of the conceptus. Since dead fetuses were macerated or partially resorbed at the time of maternal sacrifice, fetal remnants were examined only grossly. However, none of the USGS control fetuses that were alive, dead or partially reabsorbed at stages E12-E18 exhibited any signs of craniofacial maldevelopment.

**Table 4:** Development of C.B-17 SCID mouse fetuses^a^.

**USGS Litters**					
**Embryonic Stage**	**Number Litters**	**Number Fetuses**^b^	**Number Remnants**^c^	**Body Weight** **(Mean ± SE)**	**Crown-Rump Length** **(Mean ± SE)**

E12	4	32	3	0.07 g ± 0.01	8.51 mm ± 0.23
E14	5	32	5	0.20 g ± 0.01	11.04 mm ± 0.11
E16	6	19	8	0.78 g ± 0.06	17.25 mm ± 0.77
E18	18	75	15	1.00 g ± 0.03	19.08 mm ± 0.28

**MCMV Litters**					

**Embryonic Stage**	**Number Litters**	**Number Fetuses**^b^	**Number Remnants**^c^	**Body Weight** **(Mean ± SE)**	**Crown-Rump Length** **(Mean ± SE)**

E12	5	29 (11)^d^	7	0.07 g ± 0.01 (0.05 g ± 0.01)^d^	8.14 mm ± 0.24 (6.65 mm ± 0.41)^d^*
E14	5	24 (12)	10	0.18 g ± 0.01* (0.17 g ± 0.02)*	10.61 mm ± 0.20 (10.46 mm ± 0.37)
E16	6	32 (8)	9	0.51 g ± 0.02** (0.52 g ± 0.03)*	14.42 mm ± 0.23** (14.06 mm ± 0.38)*
E18	16	94 (50)	16	0.82 g ± 0.02** (0.81 g ± 0.02)*	18.05 mm ± 0.19** (18.18 mm ± 0.28)**

**a: **SCID mouse dams were intraperitoneally injected with either USGS or 10^3 ^PFU of MCMV at E4, and live fetuses were collected at the indicated embryonic (E) stages.

**b: **Live fetuses pooled across litters.

**c: **Dead/reabsorbed fetuses pooled across litters.

**d: **(fetuses nPCR MCMV DNA positive in brains and/or viscera).

Statistical significance levels (USGS vs. MCMV): * p < 0.05, ** p < 0.01, Student's *t-*tests.

Primary maternal IP inoculation with 10^3 ^PFU of MCMV at stage E4 did not adversely impact either litter size or the total number of remnants per litter (Table 4). However, maternal MCMV inoculation was associated with significant reductions in both the size and weight of the surviving fetuses. When compared to age-matched USGS controls, the live fetuses with MCMV DNA amplified in their brains and/or viscera exhibited significant reductions in their crown-rump length at E12, weight at E14, and both length and weight at E16 and E18, (Table 4; p < 0.05 or p < 0.01, Student's *t*-tests). While vertical transmission of MCMV was associated with whole body intrauterine growth retardation, none of the fetuses that amplified MCMV DNA exhibited any gross deformities (e.g., craniofacial dysmorphic features). Of note, in addition to significant reductions in body length and weight, the fetuses with nPCR amplified MCMV DNA also exhibited abnormally small head size (i.e., microcephaly). However, detailed measurements of head size were not taken at the time of sacrifice due to experimental time constraints: after the fetuses were decapitated, within 30 seconds their heads were bisected sagittally, and the right half-brains extracted from the skull and snap-frozen in liquid nitrogen for cytokine RNase protection assays (see below).

### MCMV immunohistochemistry and in situ hybridization

**Figure 2:**
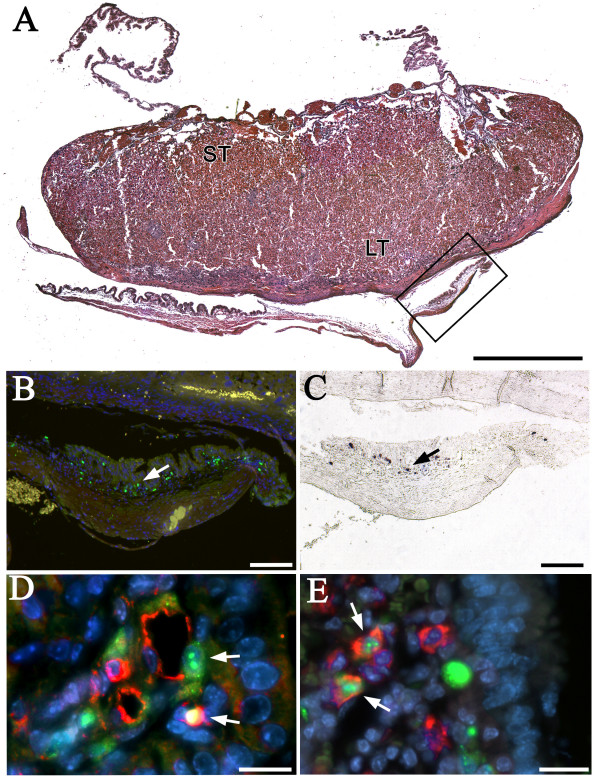
**Congenital MCMV infection in E18 fetal membranes**. Pregnant SCID mice were injected with 10^3 ^PFU of MCMV at E4. A: H&E staining of an E18 SCID mouse placenta with extraembryonic membranes. ST, spongiotrophoblastic zone; LT, labyrinthine zone; visceral yolk sac in box. B: Anti-MCMV immunostaining (green; arrow) for viral antigens in the mesodermal layer of the visceral yolk sac boxed in (A). C: In situ hybridization (purple; arrow) for MCMV RNA in the mesodermal layer of the visceral yolk sac boxed in (A). D: Anti-MCMV immunostaining (green) in endothelial cells (anti-CD31+: red) in the walls of blood vessels (arrows) within the visceral yolk sac of an E18 embryo. E: Anti-MCMV immunostaining (green, arrows) within macrophages (anti-F4/80: red) in the visceral yolk sac mesoderm of an E18 embryo. A,D&E: Bisbenzimide nuclear stain (blue). A: Bar = 1 mm; B&C: Bars = 100 μm, D&E: Bars = 20 μm.

In order to verify transplacental MCMV transmission at the cellular level, the placentas with attached extraembryonic membranes (Figure 2A) and the left half-heads from the 50 stage E18 fetuses that amplified MCMV DNA in their right half-brains or viscera were examined by immunohistochemistry (IHC; N = 50) to localize MCMV antigens, and a random sample of the 50 MCMV nPCR positive fetuses were evaluated by in situ hybridization (ISH; N = 31) to establish sites of MCMV RNA and DNA expression. MCMV was not detected by IHC or ISH in any of the USGS control E18 fetuses, or any of the left half-heads of the E18 fetuses tested that had nPCR confirmed vertical MCMV transmission. However, congenital infection was verified by IHC detection of MCMV antigens in 26% (13/50) of the fetal placentas and extraembryonic membranes from E18 fetuses that had MCMV DNA amplified by nPCR in their brains and/or viscera. In addition, MCMV RNA and DNA were identified in the placentas and extraembryonic membranes of 32% (10/31) of the MCMV nPCR positive fetuses tested. MCMV antigens (IHC: Figure 2B), and RNA and DNA (ISH: Figure 2C) were localized primarily within the fetal visceral splanchnopleuric yolk sac membranes and, less frequently, within the labyrinthine and spongiotrophoblast layers of the fetal placental disk. Double-label immunostaining confirmed congenital MCMV infection in blood vessel endothelial cells (anti-CD31, Figure 2D) and macrophages (i.e., Hofbauer cells; anti-F4/80, Figure 2E) within the mesodermal layers of the visceral yolk sac.

### Cerebral proinflammatory cytokine and cytokine receptor expression

**Figure 3:**
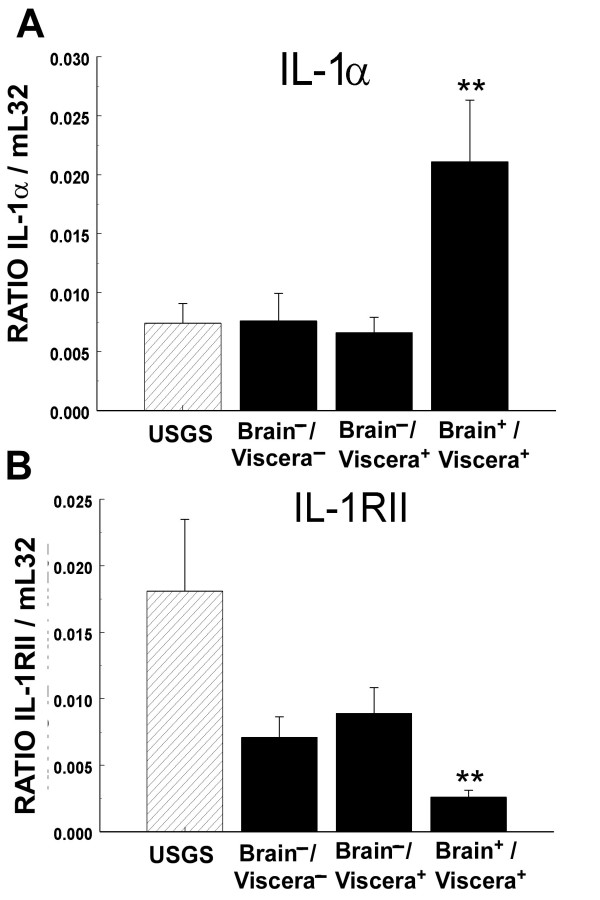
**Effects of congenital MCMV infection on cerebral IL-1α and IL-1RII transcription**. Densitometric analyses of (A) IL-1α and (B) IL-1RII mRNA expression in the brain. Pregnant SCID mice were injected at E4 with USGS or 10^3 ^PFU of MCMV and the fetuses collected at stage E18. MCMV DNA-positive fetuses were divided into three nPCR subgroups: fetuses that did not amplify MCMV DNA in either the viscera or brain (brain^-^/viscera^-^); fetuses with MCMV DNA amplified in the viscera but not the brain (brain^-^/viscera^+^); and fetuses with MCMV DNA amplified in the brain and viscera (brain^+^/viscera^+^). Autoradiographs were scanned using GelPro 3.0 software to generate maximum optical density (MOD) values, and IL-1α and IL-1RII transcript levels were normalized [(mRNA transcript)/mL32] for comparisons between autoradiographs. Statistical significance of USGS vs. MCMV nPCR subgroup comparisons: ** p < 0.01, Mann-Whitney U tests.

RNase protection assays were used to simultaneously evaluate the mRNA expression patterns for ten cytokines and six cytokine receptors (e.g., Figure 1B and 1C) in the brains of E18 control USGS (N = 28) and experimental MCMV (N = 45) group fetuses collected from SCID mouse dams IP injected at stage E4 with 10^3 ^PFU of MCMV. The MCMV group fetuses were divided into three subgroups based on their MCMV nPCR assay results: brain^-^/viscera^- ^(N = 23), brain^-^/viscera^+ ^(N = 16) and brain^+^/viscera^+ ^(all brain^+ ^fetuses were also viscera^+^; N = 6). The cytokines tumor necrosis factor-α (TNFα), transforming growth factor (TGF)-β1, TGF-β2, TGF-β3, interferon-γ (IFNγ), interleukin-1β (IL-1β), IL-1Ra, IL-12p35 and IL-12p40, as well as the cytokine receptors IL-1RI, IL-12Rβ1, IL-12Rβ2, IFNγ-Rα, IFNγ-Rβ all had similar expression levels in the brains of the USGS controls, and the experimental MCMV nPCR brain^-^/viscera^- ^and brain^-^/viscera^+ ^fetuses (all p > 0.10, Mann-Whitney U tests). In marked contrast, when compared with the control USGS group fetuses, the nPCR MCMV brain^+^/viscera^+ ^subgroup exhibited significant upregulation of cerebral IL-1α mRNA expression and significant downregulation of IL-1RII mRNA transcription (Figure 3; all p < 0.01, Mann-Whitney U test). Notably, neither the MCMV nPCR brain^-^/viscera^- ^nor the brain^-^/viscera^+ ^E18 subgroups demonstrated the same significant modulation of IL-1α and IL-1RII mRNA expression levels observed in the MCMV nPCR brain^+^/viscera^+ ^fetuses. The fact that the littermates of the E18 nPCR MCMV brain^+^/viscera^+ ^subgroup did not exhibit abnormal cerebral cytokine and cytokine receptor expression levels confirmed that the observed brain cytokine effects were related to transplacental transmission of MCMV in the fetal brain, and could not be explained by contamination of the fetus with maternal blood during specimen collection or by indirect effects secondary to maternal MCMV infection.

## Discussion

Immunodeficiencies, either innate [35,36] or acquired [37-39], are known to be detrimental to the outcome of viral infections. Notably, while immunocompetent mice are highly resistant to congenital MCMV infection [31,32], the severely T- and B-cell immunodeficient SCID mice used in this study were found to be highly susceptible to natural vertical MCMV transmission and congenital infection. Thus, during gestation the maternal immune system clearly plays a major role in controlling transplacental transmission of MCMV.

Previously, it has been hypothesized that distinctive anatomical features of the mouse placenta might contribute, in some fashion, to the obstruction of transplacental MCMV transmission [32]. Notably, the anatomy of the rodent placenta differs in a number of respects from that found in other mammalian orders. In particular, the structure of the trophoblastic layers of the murine chorioallantoic placenta is exceptional in that maternal blood is separated from fetal blood by three (hemotrichorial) trophoblast layers, a basement membrane, and a layer of fetal vascular endothelial cells. In comparison, in humans, guinea pigs and monkeys the placenta contains only a single trophoblast (monochorial) layer [32]. Thus, the two extra layers of trophoblastic cells between the maternal and fetal blood in mice could provide a physical barrier which serves to limit the vertical transfer of MCMV from the mother to the conceptus. However, since no anatomical differences between the placentas of SCID mice and congenic immunocompetent mice have been identified, and given our findings that SCID mice are highly susceptible to transplacental MCMV transmission, presumably there is some alternative mechanism, not based on the anatomy, which limits vertical MCMV transmission in immunocompetent mice. Possible candidates for an impediment to transplacental viral passage other than a physical "barrier" could include the local production of protective cytokines, the generation of intrinsic antibody, and the lack of expression of specialized cell surface receptors required for MCMV attachment and penetration of cells on one, or more, of the trophoblast layers [32]. However, whatever the barrier to transplacental virus passage in immunocompetent mice turns out to be, once it has been breached the conceptus is clearly susceptible to congenital MCMV infection [40-43].

In humans and many other mammals the fetal placenta is reportedly the initial site of congenital CMV infection [44-47]. Consequently, Fisher et al. [44] hypothesized that infection of the placenta is a necessary prerequisite for the development of congenital CMV infection in the fetus. Since human placental trophoblasts are permissive for HCMV infection *in vitro *and *in utero *[44], and given that both murine and human placentas contain similar fetal cell elements [32], we anticipated finding MCMV infection in the placentas of all of the fetuses that had vertical MCMV transmission. Instead, MCMV transcription (ISH) and translation (IHC) was detected in only 26% of the placentas and extraembryonic membranes from E18 fetuses with MCMV nPCR positive brains or viscera. However, nPCR is a more sensitive technique than IHC and ISH assays. Thus, it is possible that additional fetal placentas in this study were in fact infected with MCMV, but that the viral loads in these tissues were below the threshold of detection of our IHC and ISH assays. Alternatively, vertical MCMV transmission may have occurred without infection of the placenta, an attractive possibility given evidence that HCMV is frequently transmitted from mother to fetus by transcytosis across the syncytium without infection of syncytiotrophoblasts [44]. The level of MCMV viremia in the dams was not examined in this study, and future experiments are required to determine the extent to which transplacental MCMV transmission is dependent upon the maternal viral load.

Once transplacental MCMV transmission occurred, the fetal brain and visceral organs became prominent targets for congenital infection in SCID mice. However, the extent of intrauterine growth retardation for the fetuses with nPCR confirmed vertical MCMV transmission was not significantly different from their MCMV nPCR negative littermates (Table 4). This suggests that MCMV-induced changes in the placenta, and not direct effects of the virus on the fetus, were the primary cause of the fetal growth and developmental delays observed in the MCMV experimental groups. Since MCMV infection is known to degrade the normal tight attachments between cells *in vitro *and *in vivo *[48,49], it is possible that MCMV-induced degradation of the attachments between syncytiotrophoblasts and cytotrophoblasts in the syncytiotrophoblast layers of the placenta interfered with the normal exchange of nutrients, gasses, wastes, etc. across the maternal-fetal interface during pregnancy. Alternatively, a break-down in the attachments between syncytiotrophoblasts and cytotrophoblasts may have created channels through which cell-free virus could be transmitted from mother-to-fetus. While we did not detect any histopathological signs of placental hemorrhage, MCMV-infected endothelial cells were frequently observed in visceral yolk sac blood vessels (e.g., Figure 2D), findings which suggest that viral infection may have compromised the integrity of the maternal-fetal blood barriers. Histological evidence that congenital viral infection can induce placental pathology has been provided by Amedee et al. [50], who reported that infarcts were common at the basal plate of the placenta for rhesus macaque monkeys that developed congenital simian immunodeficiency virus (SIV) infections, and that these vascular ruptures were large enough to permit viral passage across the maternal-fetal barrier.

Another mechanism that could contribute to vertical MCMV transmission in SCID mice would be the transplacental passage of infected inflammatory cells. Since CMV is a macrophage-tropic virus [51], it has long been suspected that infected macrophages may serve as a "Trojan horse" that could convey the virus from mother-to-fetus across the placenta [50,51]. Evidence supporting a macrophage-based transplacental transport mechanism has been reported by Amedee et al. [50], who found significant reservoirs of macrophages on the maternal side of the basal placental plate, and high concentrations of Hofbauer cells (i.e., fetal macrophages) on the fetal side of the placenta in rhesus macaque monkeys that developed congenital SIV infections. The current study also detected high concentrations of MCMV-infected Hofbauer cells on the fetal side of the placenta in congenitally infected SCID mice (Figure 2E). Thus, our data are consistent with the hypothesis that maternal immunodeficiency may promote transplacental transmission of MCMV, either as free virus or contained within infected maternal macrophages, and that the virus is subsequently amplified in fetal Hofbauer cells.

The clinical correlates of transplacental MCMV transmission in SCID mice closely resembled those reported in congenital HCMV studies. Consistent with the very low rate (<0.10%) of symptomatic congenital HCMV infection in human neonates [2,3,7], we did not observe any examples where primary MCMV infection of SCID mice during pregnancy induced cytomegalic inclusion disease (CID) or any gross dysmorphic features in the fetuses. However, MCMV that crossed the placenta did exhibit a tissue tropism for the fetal SCID mouse brain, a finding in agreement with earlier clinical reports that congenital HCMV infection targets the developing brain [2,3,7] and is the leading infectious cause of human congenital CNS pathology [52-54]. Notably, in earlier experimental studies, the induction of congenital MCMV by direct inoculation of the fetus *in utero *with high-dose MCMV was found to cause CNS infection, neuronal apoptosis and gross craniofacial deformities in immunocompetent mice [40-43]. Thus, the absence of any signs of gross craniofacial or CNS malformations in the present study was not consistent with the previously reported congenital MCMV infection studies. The discrepancies between the current study and the prior investigations may be explained by the fact that direct injection of the fetus *in utero *with high-dose MCMV presumably produced significantly higher fetal viral loads than those attained by natural transplacental congenital MCMV infection in SCID mice. Consistent with the viral load hypothesis, we have observed in ongoing studies that direct injection of SCID mouse fetuses *in utero *at E11 with 68 PFU of MCMV also can induce congenital MCMV infection and neuropathology in the developing CNS (unpublished observations).

While there is an extensive literature on cerebral cytokine expression and function during viral infections of the brain in mature subjects [55], to our knowledge this was the first *in vivo *study to investigate the effects of transplacental CMV transmission on cerebral cytokine transcription. Most cytokines are expressed at low or undetectable levels in the healthy adult brain, but many are induced or modulated by CNS infections [56]. One of the most widely studied proinflammatory cytokines in the brain is IL-1. The IL-1 family is comprised of at least three proteins, the best characterized of which are IL-1α, IL-1β and IL-1 receptor antagonist (IL-1Ra). IL-1α and IL-1β are bioactive agonists, and IL-1Ra is thought to function as an endogenous receptor antagonist [56]. The biological actions of IL-1 family members are mediated by binding to two distinct forms of receptors: a biologically active type I receptor (IL-1RI) and a presumed decoy receptor IL-1RII [57]. With respect to viral infection, experimental evidence suggests that the IL-1 family plays a pivotal role in MCMV pathogenesis and the reactivation of latent virus in adult mice [58], and that IL-1 signaling in the CNS elicits responses which can either exacerbate or inhibit neuronal cell death (i.e., apoptosis) in neonatal and adult immunocompetent mice [59-61].

Notably, the E18 fetuses in this study with nPCR confirmed vertical MCMV transmission in the brain also exhibited abnormal cerebral IL-1 expression levels: IL-1α mRNA transcription was significantly upregulated and IL-1RII mRNA expression was significantly downregulated. Since IL-1α functions as an agonist and IL-RII reportedly acts as a decoy receptor that traps free IL-1α [56], the combination of increased IL-1α expression and decreased IL-1RII transcription in the brain would result in an overall net increase the amount of IL-α available for signaling in the brains of MCMV congenitally infected fetuses. Acting through a variety of different effects on the developing CNS, such as the induction of neuronal apoptosis [59-61], activation of gliosis, growth factors, adhesion molecules, corticotrophin-releasing factor (CRF), free radicals, and complement, as well as modulation cellular calcium homeostasis [56] and neuroendocrine (i.e., hypothalamic-pituitary-adrenal-axis) responses [62,63], an increase in the level of cerebral IL-1α signaling could effect the secretion of pituitary growth hormones [64], and thereby (directly or indirectly) contribute to the fetal growth retardation that is a characteristic of congenital HCMV infected humans [45,47] and MCMV infected SCID mice (this study).

The etiology of the brain pathology and sensory deficits (e.g., hearing and vision losses) induced by congenital HCMV infections is still poorly understood, and the development of a valid experimental animal model would be advantageous. Due to the species-specificity of CMV [2,15], it has not been possible to use HCMV in animal congenital infection models. As an alternative, naturally occurring transplacental viral transmission has been employed to create models of congenital HCMV infection in guinea pigs [16-24], rats [27], pigs [28] and rhesus macaque monkeys [29,30]. The rhesus macaque monkey model of congenital HCMV infection has been limited by the animal's prolonged gestation time, the fact that RhCMV is endemic throughout the available study population, as well as general considerations about working with higher primates [29,30]. To date, the most commonly used animal species employed to model congenital HCMV infections has been the guinea pig, and congenital GPCMV infection has been utilized extensively in studies by ourselves [21,22] and many others [16-20,23-26]. However, although guinea pig studies have provided significant insights into the cellular and molecular mechanisms underlying congenital GPCMV infection, the guinea pig congenital infection model is somewhat limited in that natural vertical GPCMV infection does not reproduce the CNS neuropathology that has been described in symptomatic congenital HCMV case reports [4,7-9]. Notably, a new rat model of natural transplacental RCMV transmission has recently been described, but none of the RCMV congenitally infected rat embryos or neonates reported had infectious virus, viral antigen or DNA detected in the brain [27].

Although the rate of transplacental MCMV transmission in this study reached a high of 53% at E18, and up to 21% of the fetuses collected at developmental stages E12-E18 from MCMV injected SCID mouse dams had MCMV DNA amplified in their brains, no evidence of fetal cranial deformity or CNS neuropathology was detected. Thus, while the SCID mouse model constitutes an important new experimental approach for investigating the role of maternal immunity in the development of MCMV transplacental transmission and congenital infection, at this time direct MCMV injection of the mouse fetus *in utero *remains the best available method for investigating the etiopathogenesis of congenital MCMV infection-induced birth defects [40-42]. However, there is evidence that not all of the pathologic effects of congenital CMV infection are evident at birth. Notably, there is clinical evidence that more than 25% of the children who develop congenital HCMV-induced sensory system deficits (e.g. hearing loss) do so only after the first year of life [7,10]. Because mice are extremely altricial animals, it is not until several weeks postpartum that maturation of the mouse brain neurosensory systems is comparable to that of humans at birth [65]. Thus, since that the oldest developmental age examined in the current study was E18, and given that the severity of congenital CMV pathologies can increase progressively with chronological age, it will be of considerable interest to determine in future studies whether MCMV congenitally infected SCID mice that survive beyond term birth will go on to develop late-onset sensory system (e.g., hearing and vision) deficits and CNS neuropathologies similar to those that emerge in congenital HCMV disease [4,7-9].

## Conclusion

Natural vertical transmission of MCMV occurred frequently and reproducibly in immunodeficient C.B-17 SCID mouse dams that were infected with MCMV during the first trimester of pregnancy. Kinetic studies determined that the maximum rate of MCMV transplacental transmission and congenital infection in E18 fetuses was obtained when SCID mouse dams were IP injected with 10^3 ^PFU of MCMV at embryonic stage E4. MCMV exhibited a tissue tropism for the developing fetal brain and viscera, and transplacental transmission of MCMV DNA into the fetal brain was associated with significant changes in the constitutive expression levels of the proinflammatory cytokine IL-1α and its bioactive IL-1RII receptor in the CNS. These findings have confirmed that the natural murine barrier to transplacental MCMV transmission is compromised in SCID mice, that transplacental MCMV transmission in SCID mice can induce congenital MCMV infection, and that the SCID mouse congenital MCMV infection model can provide a useful new experimental approach for future studies on the cellular and molecular mechanisms that regulate MCMV vertical transmission and viral pathogenesis.

## Methods

### Animals

Young adult (10–14 week old) specific pathogen-free C.B-17 SCID mice of both sexes were purchased from Taconic Farms (Germantown, NY). Animals were housed and maintained at the San Diego VA Veterinarian Medical Unit in microisolator cages housed in a clean room with positive air pressure and filtered air. Food, water, bedding and cages were routinely autoclaved. MCMV infected animals were housed separately from uninfected animals. Experimental protocols were approved by the San Diego VA Medical Center Animal Welfare Committee, and conformed with the PHS "Guide For the Care and Use of Laboratory Animals".

### Treatments and tissue preparation

Following established convention, pregnancies were dated as E0 on the morning of the day a vaginal seminal plug was found following overnight mating. At the designated gestational stages (E0-E7), experimental pregnant SCID mice were injected IP with 10^2^–10^4 ^PFU of MCMV in 0.2 ml of PBS (MCMV group), and control SCID mice inoculated with a similar volume of USGS diluted in PBS (USGS group). After post-injection survival to the appropriate gestational stage (E12-E18), mothers were deeply anesthetized [IP injection with 80 mg/kg sodium pentobarbital (Nembutal) and 5 mg/kg diazepam (Valium)]. All of the surgical instruments used to harvest fetuses and to dissect tissue samples were treated throughout the procedure with ELIMINase (Fisher Scientific, Pittsburgh, PA) to eliminate RNase and DNase contaminants that could degrade RNA and DNA samples or interfere with subsequent enzymatic reactions. Using a Zeiss OPMI-1 operating microscope, the peritoneal cavity was exposed by a transabdominal incision, the two horns of the uterus gently extracted, individual amniotic sacs identified and opened, and the fetuses and their placentas (with attached extraembryonic membranes) collected separately. Due to the small size of the E12 fetuses, the whole body was utilized at this stage for nPCR assay. For E14-E18 fetuses, the bodies were decapitated, the head bisected, the abdomen resected, and the abdominal viscera (i.e., the contents of the peritoneal cavity with the exception of the small intestine) and one half of the brain were processed separately for nPCR. The remaining half-brain was either snap frozen and stored at -70°C for RNase Protection Assay (RPA), or prepared for in situ hybridization (ISH) and immunohistochemistry (IHC) assays. Half-brains and placentas designated for ISH or IHC were immersion fixed overnight in 5% paraformaldehyde at 4°C [21,66], embedded in either paraffin or (cryoprotected in 30% sucrose and frozen in) OCT, sectioned (10 μm), collected onto Biobond (Ted Pella, Redding, CA) coated Fisher SuperFrost Plus slides (Fisher Scientific), and air and vacuum dried.

### Virus and cell culture

Smith strain MCMV (ATCC #VR-194) was passaged in Swiss mice. Salivary gland suspensions (10% wt/vol) were prepared in Hank's Balanced salts and 10% DMSO, aliquoted and stored at -70°C. For control inoculums, uninfected salivary gland suspensions (USGS) were prepared similarly from MCMV-free Swiss mice. MCMV infectivity was titered on subcultured mouse embryonic fibroblasts by plaque assay [67]. The titer of the MCMV stock was 3.6 × 10^6 ^PFU/ml. A fresh aliquot of stock MCMV was thawed and diluted for each experiment.

### Nested PCR for MCMV

Nested PCR (nPCR) gene amplification was conducted using two sets of 30-bp oligonucleotide primers (external and internal) selected from exon 4 of the MCMV immediate-early gene 1 from published sequence [68] as described by Collins et al. [69]. DNA was extracted from brains and viscera using the QiaAmp DNA Kit (Qiagen, Valencia, CA), as stated by the manufacturer. For the external PCR gene amplification reactions, 10 μl of sample DNA(1–3 μg), 10 μl of negative control AE Elution buffer (Qiagen), or 10 μl of positive controls [5 μl AE Elution buffer (Qiagen) plus 5 μl of purified MCMV DNA fragment Eco RI E (250 ag, 2.5 fg, 25 fg, or 250 fg), which contains the immediate-early gene 1 [68, 70]] was added to a reaction mixture containing a final concentration of 200 μM of each dNTP, 50 pmole of each external primer, 2 mM MgCl_2_, 1XGeneAmp PCR buffer II (PE Applied Biosystems, Foster City, CA), and 2 units AmpliTaq Gold (PE Applied Biosystems) in a total volume of 100 μl. Reactions were incubated at 94°C for 12 min to activate the Taq, then amplified in an Ericomp (San Diego, CA) automated thermal cycler for 35 cycles as follows: denaturation at 95°C for 40 sec, annealing of extension primers at 55°C for 40 sec, and primer extension at 72°C for 1 min, with a final extension at 72°C for 7 min.

For secondary, internal reactions, 5 μl of the primary reaction was added to a reaction mixture of 200 μM of each dNTP, 50 pmole of each internal primer, 2 mM mgCl_2_, 1XGeneAmp PCR buffer II (PE Applied Biosystems), and 2 units AmpliTaq Gold (PE Applied Biosystems) in a total volume of 100 μl. Internal reactions were incubated as in the external reactions, and amplified for 30 cycles. Amplified products were analyzed by liquid hybridization: sample mixtures [10 μl of internal reaction product + 4 × 10^5^cpm/μl ^32^P end-labeled oligo probe [69] in a final volume of 20 μl of 15 mM NaCl + 10 m MEDTA (pH 8.0)] were incubated at 100°C (5 min) to denature the amplified product, then incubated at 56°C (10 min) for annealing of the probe, loaded and run on an 8% polyacrylamide gel, and then placed under XAR-5 film (Kodak, Rochester, NY) overnight. Sensitivity of this nPCR assay was 250 ag of MCMV DNA, which corresponds to 17.5 genomic equivalents.

### Multiprobe RNase protection assay (RPA)

Total RNA was isolated from fetal brains and viscera using Trizol (Invitrogen, Carlsbad, CA), as recommended by the manufacturer. The integrity of the RNA was analyzed on agarose gels prior to RPA. RPA was performed using the RiboQuant Multi-Probe RNase Protection Assay System (BD PharMingen, San Diego, CA) according to the manufacturer's protocol. Two custom-made probe sets (BD PharMingen) contained DNA-templates for the cytokines IL-1α, IL-1β, IL-1Ra, IL-12p35, IL-12p40, IFNγ, TNFα, TGFβ1, TGFβ2, and TGFβ3, and the cytokine receptors IL-1RI, IL-1RII, IL-12Rβ1, IL-12Rβ2, IFNγRα, and IFNγRβ, as well as the housekeeping genes GAPDH and mouse large ribosomal subunit protein 32 (mL32). The DNA template sets were used to generate [α-^32^P]UTP-labeled antisense riboprobes with high specific activity by *in vitro *transcription using T7 polymerase and [α^32^P]UTP (3000 Ci/10 mCi/ml; *In Vitro *Transcription Kit, PharMingen) as recommended by the manufacturer. In brief, 10 μg of total brain RNA from each sample, Yeast tRNA (negative control), or 1 μg control RNA (PharMingen), were hybridized to 2.9 × 10^5 ^cpm/μl of labeled probe. The hybridizations were performed in eppindorf tubes, placed in a metal rack with a pressure plate (RPI Corp., Mount Prospect, IL) and submerged in a water bath overnight at 56°C. Samples were then digested with RNase, treated with proteinase K, ethanol precipitated and resuspended in 5 μl of loading buffer (Pharmingen). Protected fragments were purified and separated on 5% polyacrylamide gels, dried, and put under XAR-5 film (Kodak) for 3–4 day exposures. The films were scanned (Hewlett-Packard ScanJet 4C, Palo Alto, CA), and the resulting bands quantified using Gel Pro (Media Cybernetics, Silver Spring, MD) Maximum Optical Density (MOD) settings. For each sample lane, the intensity of the cytokine and cytokine receptor bands were normalized by dividing their MOD values by the corresponding mL32 housekeeping gene MOD values.

### MCMV in situ hybridization (ISH)

In situ hybridization for MCMV mRNA and DNA was conducted with 1 μg of an equimolar mixture of MCMV fragments EcoR1-E, EcoR1-V and EcoR1-P (kindly provided by Dr. Deborah Spector) which correspond to regions of the MCMV genome where transcription occurs primarily at immediate-early, early and late times during infection respectively [67,70,71], or the vector pACYC (used as a negative control), were nick translated in the presence of 40 μM dCTP, dATP, dGTP, DIG-11-dUTP, 10 mM DTTP for 1.5 h using a Invitrogen Nick Translation kit (Invitrogen). The reactions were stopped, precipitated and resuspended in 100 μl of 10 mM Tris in 1 mM EDTA (pH7.5). Tissue sections (10 μm), prepared from paraffin [deparaffinized in Hemo-De (Fisher Scientific, Pittsburgh, PA) and rehydrated in graded ethanol] or OCT frozen blocks, were placed in 1×PBS twice for 5 min. For prehybridization, sections were placed in 0.2 N HCl at room temperature for 20 min, dipped in ddH_2_O, put in 2×SSC at 70°C for 30 min, dipped in ddH_2_O, digested in 20 mM Tris (pH7.4), 2 mM CaCl_2_, 4 μg/ml proteinase K at 37°C for 30 min, washed in ddH_2_O at room temperature for 5 min, dehydrated in graded alcohol's, and then air dried. Hybridization buffer consisted of 50% de-ionized formamide, 10% dextran sulfate, 0.05% polyvinylpyrolidone, 50 mM Tris-HCL (pH7.4), 300 mM NaCl, 250 μg/ml herring sperm DNA, 500 μg/ml mouse liver RNA, 100 μg/ml Poly A, and 0.05% SDS. To detect MCMV DNA and mRNA simultaneously, sections with hybridization buffer were put at 95°C for 6 min, then on ice, sealed with DPX (Fluka, Milwaukee, WI) and hybridized at 42°C overnight with either the E, P, and V mixture, or the pACYC (control) labeled probe (33 pg/μl resuspended in hybridization buffer). Sections were then washed sequentially in 2×SSC+0.1% Triton X-100 (1 h at room temperature), 1×SSC+0.1% Triton X-100 (1 h at room temperature), 0.5×SSC+0.1% Triton X-100 (30 min at 37°C), 0.1×SSC+0.1% Triton X-100 (1 h at room temperature), and finally 1×Washing buffer (100 mM Tris, 150 mM NaCl pH7.5)+0.5% Triton X-100 (10 min at room temperature. After blocking (2% Roche blocking buffer (Roche, Indianapolis, IN) in Washing buffer plus 0.5% Triton X-100; 30 min at room temperature), sections were incubated with a 1:750 dilution of anti-digoxigenin antibody (Roche) in 1% Blocking buffer (2% Roche Blocking buffer diluted 1:1 with Washing buffer) (2 h at room temperature). Sections were then washed 3× in Washing buffer containing 0.5% Triton (5 minutes each at room temperature), followed by an equilibration in Detection buffer (100 mM Tris, 100 mM NaCl pH9.5), 10 mM MgCl_2_, and 240 μg/ml levamisol (2 min at room temperature). To visualize the digoxigenin reaction development, sections were incubated with 0.33 mg/ml NBT, 0.16 mg/ml BCIP in 1×Detection buffer+10 mM MgCl_2_+240 μg/ml levamisol (overnight at room temperature in the dark). Development was stopped by washing the slides 2×ddH2O (5 minutes each), and cover slipped with Crystalmount (Biomeda Corp., Foster City, CA).

### MCMV immunohistochemistry (IHC)

In brief, an enzymatic unmasking of antigenic sites was performed by treating sections with 0.1% w/v trypsin in PBS for 30 min at 37°C. Nonspecific binding was blocked using a M.O.M (Mouse-On-Mouse) kit (Vector Labs, Burlingame, CA) as described by the supplier, incorporating 5% BSA and 0.1% Triton-X100 in PBS in the initial blocking step. Sections were then incubated overnight at 4°C with mouse anti-MCMV (see below) and either rat anti-F4/80 (anti-macrophage/microglia: Serotec, Raleigh, NC) or rat anti-CD31 (anti-endothelial cell: Pharmingen, La Jolla, CA). Subsequently, sections were incubated at room temperature for 1 h with Alexa Fluor^tm ^488 goat anti-mouse IgG (Molecular Probes, Eugene, OR), and then 1 h with Alexa Fluor^tm ^594 goat anti-rat IgG (Molecular Probes). Control tissues were incubated with normal mouse IgG (for anti-MCMV) and normal rat IgG (for F4/80 and CD31), and processed as above.

Anti-MCMV hyperimmune sera was prepared as described by Inada et al. [72]: Specific Pathogen Free Charles Rivers CD1 mice were IP injected with 10^4 ^PFU of MCMV, followed by two further MCMV IP inoculations at 2-week intervals, and then bled 7 days after the last injection. IgG Fc fragments were isolated from sera using an Immunopure-G^® ^IgG purification kit (Pierce, Rockford, IL), and protein concentration determined using a BAC Protein Assay kit (Pierce).

### Photomicroscopy

Images were obtained using a Zeiss Axioplan 2 microscope equipped with fluorescent illumination and fluorescein, Texas Red, and DAPI filter sets. Images were digitally captured using an KX85 CCD camera (Apogee Instruments Inc., Tuscon, AZ), and optimized in Image Pro (Media Cybernetics) or Adobe Photoshop (San Jose, CA).

### Statistical analyses

All data are expressed as the mean ± SE, and differences between groups were evaluated by Student's *t*-tests and Mann-Whitney U tests using SPSS (SPSS Inc., Chicago, IL), with probability values p < 0.05 considered significant.

## List of abbreviations

Cytomegalic inclusion disease (CID); cytomegalovirus (CMV); central nervous system (CNS); embryonic stage (E); Glyseraldehyde-3-phosphate dehydrogenase (GAPDH); hematoxylin and eosin (H&E); immunohistochemistry (IHC); in situ hybridization (ISH); interferon (IFN); interleukin (IL); intraperitoneal (IP); intrauterine growth retardation (IUGR); Maximum Optical Density (MOD); nested polymerase chain reaction (nPCR); mouse large ribosomal subunit protein 32 (L32); plaque forming units (PFU); RNase Protection Assay (RPA); sensorineural hearing loss (SNHL); severe combined immunodeficient (SCID); simian immunodeficiency virus (SIV); transforming growth factor (TGF); tumor necrosis factor (TNF); uninfected salivary gland suspension (USGS).

## Competing interests

The author(s) declare that they have no competing interests.

## Authors' contributions

NKW conceived of the study, was responsible for the experimental design, performed all experiments and drafted the manuscript. DVJ participated in carrying out the experiments and assisted with the virus preparation, cell culture, ELISA, nPCR, RPA and *in situ *hybridization assays. FJK participated in carrying out the experiments and assisted with the tissue processing, immunohistochemistry, photomicroscopy, nPCR assays and statistical data analyses. All authors read and approved the final manuscript.
